# Tumor resident memory CD8 T cells and concomitant tumor immunity develop independently of CD4 help

**DOI:** 10.1038/s41598-023-33508-1

**Published:** 2023-04-18

**Authors:** Terry R. Medler, Gwen Kramer, Shelly Bambina, Andrew J. Gunderson, Alejandro Alice, Tiffany Blair, Lauren Zebertavage, Thomas Duhen, Rebekka Duhen, Kristina Young, Marka R. Crittenden, Michael J. Gough

**Affiliations:** 1 Earle A. Chiles Research Institute, Robert W. Franz Cancer Center, Providence Portland Medical Center, NE Glisan St., Portland, OR 480597213 USA; 2 The Oregon Clinic, Portland, OR 97213 USA; 3 Present Address: Pelotonia Institute for Immuno-Oncology, The Ohio State University Comprehensive Cancer Center – Arthur G. James Cancer Hospital and Richard J. Solove Research Institute, The OH State University, Columbus, OH 43210 USA

**Keywords:** Cancer microenvironment, Tumour immunology, Tumour immunology

## Abstract

Tissue resident memory (Trm) CD8 T cells infiltrating tumors represent an enriched population of tumor antigen-specific T cells, and their presence is associated with improved outcomes in patients. Using genetically engineered mouse pancreatic tumor models we demonstrate that tumor implantation generates a Trm niche that is dependent on direct antigen presentation by cancer cells. However, we observe that initial CCR7-mediated localization of CD8 T cells to tumor draining lymph nodes is required to subsequently generate CD103^+^ CD8 T cells in tumors. We observe that the formation of CD103^+^ CD8 T cells in tumors is dependent on CD40L but independent of CD4 T cells, and using mixed chimeras we show that CD8 T cells can provide their own CD40L to permit CD103^+^ CD8 T cell differentiation. Finally, we show that CD40L is required to provide systemic protection against secondary tumors. These data suggest that CD103^+^ CD8 T cell formation in tumors can occur independent of the two-factor authentication provided by CD4 T cells and highlight CD103^+^ CD8 T cells as a distinct differentiation decision from CD4-dependent central memory.

## Introduction

To safely generate long-lived CD8 T cell memory responses, the immune system applies a form of two-factor authentication, where both CD4 and CD8 T cells need to recognize distinct antigen epitopes in the same pathogen. This signal integration occurs at the level of the dendritic cell in draining lymph nodes, where the classic three cell model dictates that CD4 T cells license dendritic cells via CD40-CD40L interactions to optimally generate T central memory (Tcm) CD8 T cells to cross-presented antigen^1–3^. Trm represent an alternative population of memory CD8 T cells that remain resident in tissues and in tumors. These have several critical features often including expression of CD103, which is TGFb-inducible and can bind E-cadherin to form a T cell-epithelial cell niche; and an activation marker such as CD69^4^. CD69 can be induced in the tumor environment independent of cognate antigen^5^ and inflammation-induced CD69 can help retain T cells in tissue or lymph node sites via suppression of S1PR1-mediated tissue exit signals^6^. In tumors, chronic antigen exposure also results in expression of additional activation markers such as CD39^7,8^. Low numbers of CD103^+^CD39^+^ CD8 T cells in tumors correlates with poor responsiveness to conventional cancer therapies in patients^7^. We recently demonstrated that blocking implantation immunity^9,10^ using anti-CD40L antibodies blocked development of an intratumoral antigen-specific CD8 T cell population with a CD103^+^CD39^+^ Trm phenotype^11^. Using this information, we aim to understand the mechanisms by which Trm form to help understand how to generate more of these cells in poorly infiltrated tumors to improve outcomes for poorly responsive patients^12^.

Much of our prior work depended on the Panc02 cell line, which was generated by MCA-mutagenesis of mouse pancreas^13^. The Panc02-SIY cell line engineered to express the model antigen SIY is a useful tool to monitor a tumor antigen-specific immune response over the course of therapy^14–17^. However, the Panc02 cell line has neither the Kras nor p53 mutations characteristic of pancreatic adenocarcinoma in humans^18^. The *Pdx1-Cre*^+*/−*^* Kras*^*LSL-G12D/*+^ *Trp53*^*LSL-R172H/*+^ (KPC) genetically engineered mouse model (GEMM) results in aggressive tumorigenesis with authentic pancreatic histology^19^. Tumor progression in the pancreas is accompanied by progressive infiltration of macrophages, systemic myeloid dysregulation, but devoid of T cell involvement^20^. As a result of the dominant driver mutations, GEMM and GEMM cell lines can have orders of magnitude fewer mutations than human cancer cells^21^, and do not share target antigens between lines to allow for comparative studies.

In this study, we generated a pancreatic cancer GEMM incorporating the model tumor antigen SIYRYYGL (SIY) and a panel of GEMM-derived pancreatic cell lines with authentic and varied pathology for use in orthotopic and subcutaneous settings. Using these models, we demonstrate that to form a CD103^+^ CD8 T cell niche in tumors requires direct antigen presentation by cancer cells through MHC class I. Despite this direct interaction in the tumor environment, we show that CD8 T cells require initial CCR7-mediated localization to lymph nodes to subsequently form a tumor CD103^+^ CD8 T cell population. Initial responses that generate CD103^+^ CD8 T cells are dependent on CD40L signals but independent of CD4 T cell help, and we show that the CD8 T cells can provide their own CD40L to allow CD4 independent CD103^+^ CD8 T cell differentiation. We demonstrate that CD8 T cells induced by tumor implantation are superior to CD8 T cells induced by a vaccine at preventing secondary tumor growth, and that this concomitant tumor immunity is dependent on CD40L and can occur independent of CD4 T cells. These data highlight the distinct differentiation triggers that support Trm differentiation versus conventional Tcm differentiation and suggest that Trm-focused vaccines may be a better option to generate protective anti-tumor immunity.

## Results

### Generation of cell lines derived from KPC and KPC-LSIY pancreatic adenocarcinoma

**Figure 1:**
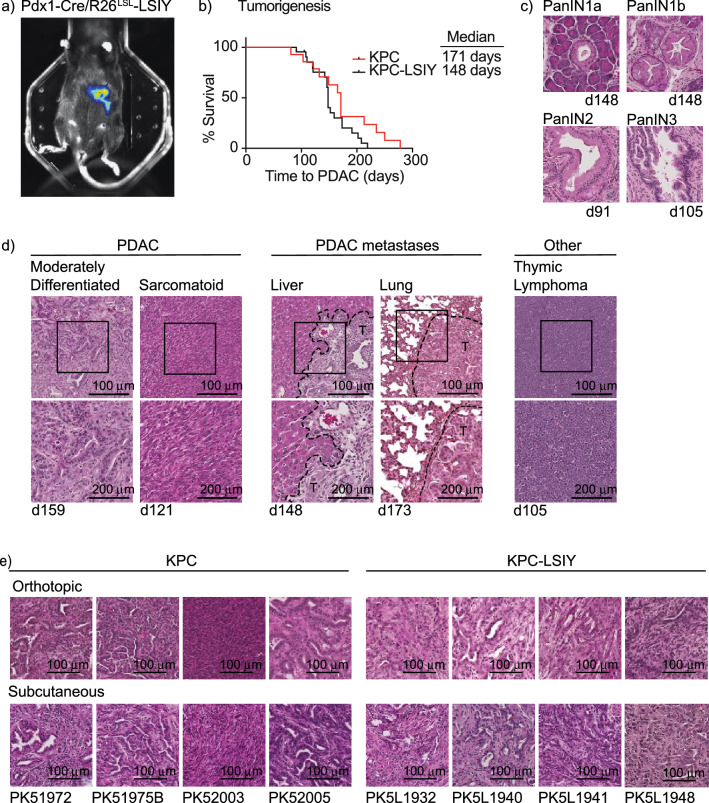
Characterization of tumors in KPC-LSIY mice. (**A**) In vivo luciferase expression in Pdx1-cre + /LSIY + by MuriGlo-assisted CT scan. (**B**) Survival curves of KPC mice compared to KPC-LSIY mice. (**C**) Representative histology of premalignant progression in KPC-LSIY mice. (**D**) Representative histology of moderately differentiated and sarcomatoid PDAC phenotypes, liver metastasis, lung metastasis, as well as thymic lymphoma and in KPC-LSIY mice. (**E**) Representative histology of orthotopic and subcutaneous tumors using KPC (PK5) and KPC-LSIY (PK5L)-derived tumor cell lines.

To generate pancreatic-specific expression of the model antigen SIY, we crossed *Pdx1-Cre* to *R26*^*LSL-LSIY*^ mice and generated mice homozygous for expression of both alleles*.* The luciferase-SIY fusion protein ensures cytoplasmic expression of the model tumor antigen as opposed to alternatives such as expression of whole ovalbumin, which results in a secreted protein antigen^22^ with the potential to compromise studies of tumor antigen cross-presentation. Bioluminescent imaging demonstrated in vivo luciferase expression was restricted to the pancreas in these mice (Fig. 1a). To generate spontaneous pancreatic tumors in these mice, we crossbred *Pdx1-Cre/R26*^*LSL-LSIY*^ with *Kras*^*LSL-G12D/*+^*Tp53*^*LSL-R172H/*+^ mice to generate KPC-LSIY mice as well as with *Pdx1-Cre* mice to generate conventional KPC mice. We found no significant change in neoplastic progression to PDAC between KPC and KPC-LSIY mice (Fig. 1b), with evidence of all stages of premalignant PDAC progression in KPC-LSIY mice (Fig. 1c), as has been reported for KPC mice^19^. We found evidence of metastasis to both the liver and lung, albeit at lower rates than was reported in KPC mice^19^ (Table 1 and Fig. 1d). However, metastases rates may be underreported, as we collected tissues for histologic confirmation when there was visual evidence of macrometastases in these tissues. We also observed metastases to distant lymph nodes, including to the axillary and mediastinal lymph nodes, as previously reported in KPC mice^23^ (Table 1). As with KPC mice, we similarly found occasional thymic lymphomas in KPC-LSIY mice (Table 1 and Fig. 1d). Pancreatic tumors were frequently large and firm, with ascites often observed prior to necropsy. Histologically, PDACs in KPC and KPC-LSIY mice were largely similar, with moderate-to-poor differentiation representing the most common phenotype. There was also occasional evidence of intestinal blockage, biliary obstruction, and hepatic necrosis, as is observed in KPC mice^19^ and human patients. Together, these data demonstrate that expression of a fusion protein of luciferase and SIY does not impact authentic tumor progression in this GEMM model.

**Table 1:** Characteristics of KPC-LSIY mice.

ID	Age (d)	PDAC	Histology	Metastases		Other
				Liver	Lung	
1	159	Yes	Glandular	Yes	Yes	
2	121	Yes	Glandular	No	No	
3	121	Yes	Sarcomatoid	Yes	No	
4	91	No	PanIN	No	No	
5	148	Yes	Glandular	No	No	
6	148	Yes	Glandular	No	No	
7	105	Yes	Glandular	No	No	Thymic Lymphoma
8	108	Yes	Glandular/Poorly differentiated	No	No	
9	173	Yes	Glandular	No	No	
10	173	Yes	Glandular	No	Yes	
11	91	Yes	Glandular	No	No	Axillary LN
12	110	Yes	Glandular	Yes	No	
13	202	Yes	Glandular	No	No	
14	152	Yes	Glandular	Yes	No	
15	142	Yes	Glandular	No	No	
16	219	Yes	Glandular	No	No	
17	146	Yes	Glandular	No	No	
18	146	Yes	Glandular	No	No	
19	148	Yes	Glandular	No	No	
20	191	Yes	Glandular	No	No	Mediastinal LN
21	208	Yes	Sarcomatoid	No	No	
22	148	Yes	Glandular	Yes	No	Mesentery
				5/21 (23.8%)	2/21 (9.5%)	

To evaluate whether antigen-specific responses to the expressed model antigen can be generated in tumor-bearing mice, we evaluated the response to a strong vaccine also bearing the model antigen. We generated *Pdx1-Cre/R26*^*LSL-LSIY*^, KPC, and KPC-LSIY mice and vaccinated them with *Listeria monocytogenes* (*Lm*) expressing the relevant model antigen SIY (*Lm-SIY*), or the irrelevant model antigen Ovalbumin (*Lm-Ova*). As an additional control, we also tested the response in POET mice that express ovalbumin in the prostate^24^. Vaccination with SIY-expressing *Lm* (*Lm-*SIY) led to a large SIY-specific CD8^+^ T cell response 7d post-vaccination in wt and POET mice, which was absent in *Pdx1-Cre/R26*^*LSL-LSIY*^ and KPC-LSIY mice (Supplemental Fig. S1a). Similarly, *Lm-Ova* vaccination significantly expanded SIINFEKL-reactive CD8^+^ T cells in wt, *Pdx1-Cre/R26*^*LSL-LSIY*^, and KPC-SIY mice, but not POET mice (Supplemental Fig. S1b). These data suggest that as in POET mice expressing ovalbumin in the prostate, mice expressing SIY in the pancreas are tolerant to this model antigen, but otherwise exhibit functional antigen-specific immunity. This is consistent with prior reports using the *R26*^*LSL-LSIY*^ mice which demonstrated central thymic tolerance to the model antigen SIY ^25^. To attempt to overcome tolerance, we tested a homologous prime/boost strategy with *Lm*. As expected, we observed an increase in IFN$\upgamma$^+^ SIY-reactive CD8^+^ T cells in wt and POET mice 7d post-boost with *Lm-SIY*, and an increase in IFN$\upgamma$^+^ SIINFEKL-reactive CD8^+^ T cells in wt and *Pdx1-Cre/R26*^*LSL-LSIY*^ mice 7d post-boost with *Lm-Ova* (Supplemental Fig. S1c-d). However, mice expressing LSIY remained unresponsive to SIY vaccination. Together, these data indicate that mice containing the *Pdx1-Cre/R26*^*LSL-LSIY*^ allele, including KPC-LSIY mice are tolerant to SIY.

Due to antigen-specific tolerance the KPC-LSIY GEMM is restricted in its usefulness to study antigen specific T cell responses in vivo; however, cell lines generated from these tumors have the potential to be a valuable resource for evaluating T cell-based therapies. Importantly, cell lines derived from GEMM retain authentic driver mutations and responses to treatments^26^, and provide multiple levels of convenience by permitting orthotopic or subcutaneous implantation in mice bearing targeted knockouts to understand their influence on tumor growth and response to treatment. We therefore isolated cell lines derived from KPC and KPC-LSIY mice (termed PK5 and PK5L, respectively) to provide a novel resource for therapeutic studies using GEMM-derived tumors. Histological evaluation of implanted PDAC cell lines derived from KPC and KPC-LSIY mice revealed that most tumors were moderately differentiated, except for PK52003 resembling a poorly differentiated phenotype with sarcomatoid features, and the different tumors were consistent in their histology whether implanted subcutaneously or orthotopically (Fig. 1e).

### Cells with a CD103^+^CD39^+^ phenotype in tumors actively engage cognate antigen in the tumor

**Figure 2:**
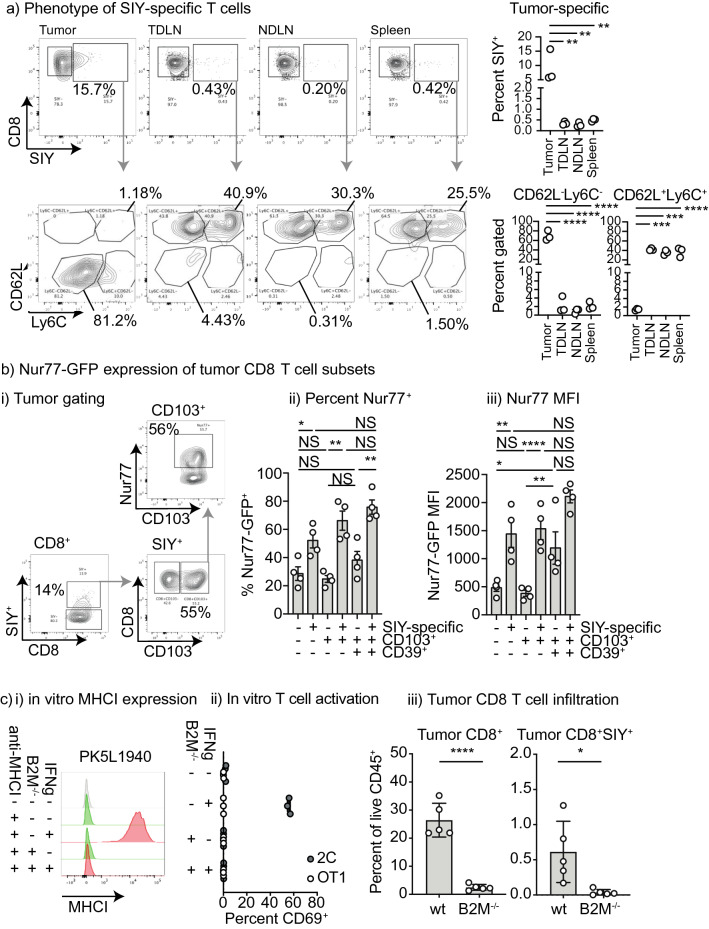
Direct interaction of tumor antigen specific T cells with antigen in tumors. (**a**) Analysis of CD8 T cells in tumor and peripheral lymphoid organs. PK5L1940 tumors were implanted sc. into C57BL/6 mice and tumors, tumor-draining lymph node (TDLN), non-draining lymph node (NDLN), and spleen were analyzed for SIY-specific CD8 T cells at d14. Subplots show CD62L and Ly6C expression on SIY-specific CD8 T cell populations in the lymphoid organs versus the tumor. (**b**) PK5L1940 tumors were implanted sc. into Nur77-GFP mice and tumor infiltrating T cells were analyzed by flow cytometry. (i) representative flow plots. The SIY-specific T cell population of infiltrating CD8 T cells was identified by pentamer staining, and CD103 + and CD103 + CD39 + subpopulations were identified and Nur77-GFP expression was determined in each cell type. Graphs show (ii) Percent Nur77-GFP + and (iii) MFI of Nur77-GFP in each group. (**c**) PK5L1940 cancer cells were deleted of B2M expression using CRISPR/Cas9. (i) MHCI expression in cancer cells was detected by flow cytometry compared to isotype control antibody (gray), with cells untreated (green) or pre-treated with IFNg (red). (ii) PK5L1940 or PK5L1940-B2M^−/−^ were co-cultured with SIY-specific 2C cells or Ova-specific OT1 cells and assessed for expression of the activation marker CD69. (iii) PK5L1940 or PK5L1940-B2M^−/−^ were implanted sc. into C57BL/6 mice and harvested at d14 and analyzed for infiltrating CD8 T cells and SIY-specific CD8 T cells by flow cytometry. Key: NS = not significant; * = p < 0.05; ** = p < 0.01; *** = p < 0.001; **** = p < 0.0001. Results are representative of two or more experiments.

To understand the distribution and phenotype of tumor-specific T cells following implantation of cell lines derived from Pdx-Cre^+/−^ Kras^(G12D)+/−^ Trp53^(R172H)+/−^ Luciferase-SIY^+^ tumors in mice, we performed multiparameter flow cytometry for SIY-specific T cells in PK5L1940 tumors, TDLN, NDLN, and spleen to look for low incidences of antigen-specific T cells. As expected, SIY-specific T cells were enriched in the tumor compared to all other sites, but small residual populations of SIY-specific T cells could be found distributed around the animal representing 0.2–1% of CD8 T cells (Fig. 2a), and such cells were undetectable in non-tumor-bearing mice (Supplementary Fig. S2a). Studies have demonstrated that recirculating central memory T cells are CD62L^+^ and Ly6C expression enriches for lymph node homing central memory T cells^27,28^. In the tumor, SIY-specific CD8 T cells are highly enriched for a CD62L^−^Ly6C^−^ population while the peripheral SIY-specific T cells have a mixture of Ly6C^−^CD62L^+^ and Ly6C^+^CD62L^+^ cells consistent with a range of central memory T cells phenotypes recirculating through secondary lymphoid organs (Fig. 2a). Similarly, the CD8 T cells that are not SIY-specific exhibit similar differentiation patterns in the tumor versus the secondary lymphoid organs (Supplementary Fig. S2b). These data indicate that while tumor-specific T cell numbers are higher in the tumor, there remains a circulating population of memory phenotype tumor-specific T cells in mice bearing PK51940 tumors derived from Pdx-Cre^+/−^ Kras^(G12D)+/−^ Trp53^(R172H)+/−^ Luciferase-SIY^+^ mice.

To identify T cells with Trm phenotypes and characterize their interaction with antigen in the tumor environment, we profiled the T cells for expression of CD103 and CD39. While this is not an exhaustive profile, it matches the phenotype of tumor antigen-reactive T cells that accumulate in human tumors^7^. To determine the degree of ongoing antigen-specific interaction among cells with this phenotype in the tumor, we implanted tumors in Nur77-GFP^+^ mice. Nur77 is upregulated 4–12 h following antigen recognition in CD8 T cells and in Nur77-GFP^+^ mice GFP expression can be used as an indicator of antigen recognition^29^. While Nur77-GFP is upregulated transiently on T cell receptor activation^29^, in tumors Nur77-GFP + cells are representative of T cells with both a recent and functional response to TCR ligation^5^, enriching for tumor antigen-reactive T cells. In SIY pentamer positive cells, the percentage Nur77-GFP positive and the MFI of Nur77-GFP is significantly higher than that seen in the SIY-pentamer negative cells (Fig. 2bi–iii). Among SIY-pentamer-binding T cells in untreated PK5L1940 pancreatic adenocarcinomas, approximately half of the CD103^+^ T cells express Nur77-GFP (Fig. 2bi–ii, Supplemental Fig. S3a), indicating that these Nur77-GFP^+^ CD103^+^ CD8 T cells are actively engaging antigen. In both SIY-specific CD8 T cells and CD8 T cells of unknown specificity, the highest proportion expressing Nur77-GFP and highest Nur77-GFP MFI is observed in the CD103^+^CD39^+^ double positive cells (Fig. 2bii–iii). Given that prior data indicates that Nur77 induction correlates with TCR affinity^29^, this data is consistent with studies showing that Trm have higher antigen affinity^8,30^. Higher Nur77-GFP expression among tumor infiltrating Trm is not limited to the PK5L1940 model as the same patterns are also observed in Panc02 pancreatic adenocarcinomas expressing the model antigen SIY (Supplemental Fig. S3b). These data confirm that CD103^+^CD39^+^ CD8 T cells that share features with Trm^7^ in murine pancreatic tumors are actively engaging with antigen in the tumor environment.

CD103 expression can allow T cells to directly interact with normal cells or cancer cells expressing E-cadherin, allowing close locoregional interactions with cancer cells which can participate in immune surveillance of tumors^31^. To understand whether antigen-specific interactions of CD103^+^ CD8 T cells are with cancer cells or with antigen-presenting cells in the tumor stroma, we manipulated MHCI expression by the cancer cells. While the PK5L1940 pancreatic adenocarcinoma cells have a low basal level of MHCI, MHCI can be upregulated via exposure to IFN (Fig. 2c), meaning that CD8 T cells may have varying TCR activation in a microenvironmental niche according to fluxes in IFN levels. To block direct presentation, we used CRISPR to delete B2M from the cancer cells to generate cancer cells that were unable to express MHCI even following IFNg stimulation (Fig. 2ci), and these cells were unable to directly present antigen to tumor-specific CD8 T cells in vitro (Fig. 2cii). Tumors lacking B2M grew moderately faster than wt tumors (mean tumor weight at d14 PK5L1940 42.8 mg PK5L1940 B2M^−/−^ 90.8 p < 0.01) suggesting that although wt tumors have low basal MHCI, CD8 T cell immunosurveillance regulates tumor growth in this model. Notably, the tumors lacking MHCI exhibited deficient CD8 T cell infiltrates, resulting in a deficiency in SIY-specific T cells (Fig. 2ciii). CD8 T cell numbers in the peripheral blood are not impacted by loss of MHCI expression in cancer cells (mean CD8 T cell number/µl peripheral blood d7 PK5L1940 1010 CD8/µl PK5L1940 B2M^−/−^ 1024 CD8/µl p = 0.87), indicating that there is no systemic loss of CD8 T cells in these mice. These data indicate that a full T cell niche does not form without direct presentation in the tumor environment. These data also indicate that although the cancer cells have low baseline antigen presentation, the ability to regulate direct antigen presentation is essential to sustain T cell infiltrates.

### T cell entry to lymph nodes is required to form CD103^+^ CD8 T cells in tumors

**Figure 3:**
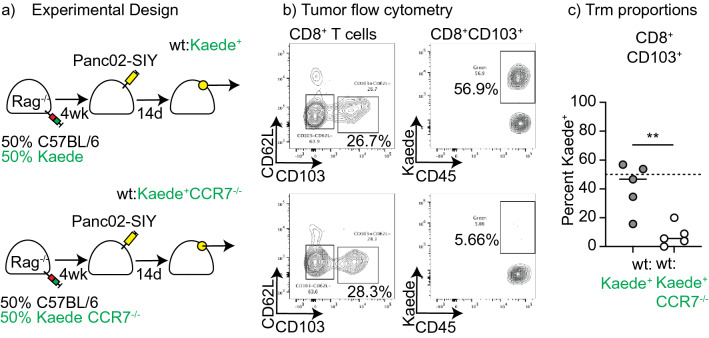
Role of direct presentation in Trm formation. (**a**) Rag^−/−^ mice were reconstituted with a 1:1 mix of splenocytes from wt and Kaede mice, or a 50:50 mice of splenocytes from wt and Kaede CCR7^−/−^ mice. Following reconstitution, mice were implanted sc. with tumors and tumor-infiltrating CD8^+^CD103^+^ T cells were analyzed for Kaede expression after 14 days. (**b**) Representative gating on CD103^+^ T cells, and Kaede expression by these cells. (**c**) Summary of percent of CD103^+^ cells that are Kaede^+^ in mice given Kaede or Kaede CCR7^−/−^ cells. Key: ** = p < 0.01. Results are representative of two or more experiments.

In theory, these data could suggest CD103^+^ CD8 T cells emerge from direct interactions with cancer cells; however, cross-presentation of tumor antigen by key dendritic cell populations has been shown to be essential for T cell responses to tumors^32^. For this reason, we designed experiments to understand whether CD103^+^ CD8 T cells arise from interactions with antigen presenting cells in lymph nodes. Naïve CD8 T cells depend on CCR7 to localize to dendritic cells in lymph nodes to initiate immune responses^33^. Total CCR7^−/−^ mice have defective lymph nodes^33^, so to overcome this we reconstituted Rag^−/−^ mice with a 1:1 mix of splenocytes from wild type mice and splenocytes from either Kaede mice, or Kaede CCR7^−/−^ mice. The Kaede transgene has green fluorescence^34^ and allows us to distinguish the lymphocytes that develop from these populations. Host Rag^−/−^ mice only lack B and T lymphocytes and the genotype of the host myeloid population including dendritic cells is unchanged between the differently reconstituted groups. Splenocyte reconstitution of Rag^−/−^ mice results in non-specific homeostatic expansion resulting in most T cells exhibiting a memory or effector phenotype^35^, and APC from the Rag^−/−^ host will have unimpaired function. This ensures that in the reconstituted Rag^−/−^ mice there are memory and effector populations that can both directly enter lymph nodes and directly enter tissues, potentially encountering antigen in each location. Following reconstitution, we observed no significant difference in the proportion of blood CD8^+^ T cells with the Kaede transgene between wt:Kaede and wt:Kaede CCR7^−/−^ mice (mean CD8 T cell proportion Kaede^+^ in the peripheral blood at week 4 wt:Kaede 45.86% wt:Kaede CCR7^−/−^ 37.8% p = 0.45). Reconstituted mice were challenged with Panc02-SIY tumors and tumor-infiltrating cells were analyzed by flow cytometry (Fig. 3a). If the Kaede cells were no different in their ability to form CD103^+^ CD8 T cells, then we would expect approximately 50% of the CD103^+^ CD8 T cells expressing the Kaede transgene. While the proportion of CD8 T cells and CD103^+^ CD8 T cells in the tumors were not different between wt:Kaede and wt:Kaede CCR7^−/−^ mice, the proportion of CD103^+^ CD8 T cells with the Kaede transgene were significantly different (Fig. 3b-c). Approximately half of the CD103^+^ CD8 T cells expressed the Kaede transgene in the wt:Kaede mice, but few expressed the Kaede transgene in the wt:Kaede CCR7^−/−^ mice. The numbers of SIY-specific CD103^+^ CD8 T cells infiltrating tumors were very low, but were also divergent in their distribution between the between Kaede and Kaede CCR7^−/−^ cells (mean of 79.5% vs 9.7% p < 0.05). These data indicate that despite the need for ongoing direct antigen engagement in the tumor to sustain CD103^+^ CD8 T cells (Fig. 2c), the tumor-specific T cells depend on lymph node entry via CCR7 for the interactions that result in CD103^+^ CD8 T cell formation.

### Role of CD4 T cells in tumor-specific CD103^+^ CD8 T cell formation

**Figure 4:**
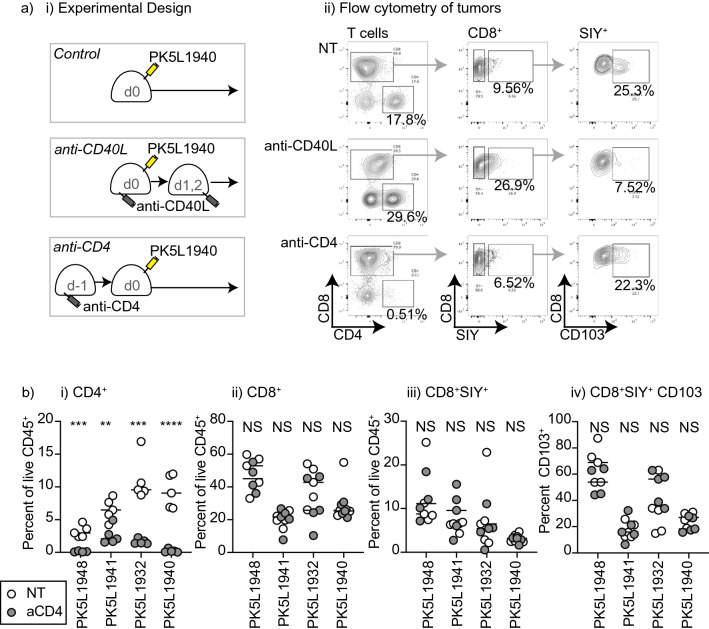
CD4 depletion minimally impacts CD8 T cell responses to tumor implantation. (**a**) PK5L1940 pancreatic adenocarcinoma cells were injected sc. into C57BL/6 mice, C57BL/6 mice treated with anti-CD40L on d0, 1, and 2, or C57BL/6 mice treated with anti-CD4 on d-1. ii) Representative flow cytometry of tumor infiltrating T cells 14d following implantation. Plots show gated CD90.2^+^ T cells, and subgates for CD8^+^ T cells, and SIY-pentamer^+^ CD8 T cells. (**b**) Summary of T cell infiltration of PK5L1948, PK5L1941, PK5L1932, and PK5L1940 tumors derived from Pdx-Cre+/− Kras(G12D)+/− Trp53(R172H)+/− Luciferase-SIY + mice implanted sc. into C57BL/6 mice or C57BL/6 mice treated with anti-CD4 on d-1. Graphs show tumor the proportion of CD4, CD8, CD8 SIY-specific T cells, and CD103 expression on CD8 SIY-specific T cells. Key: NS = not significant; * = p < 0.05; ** = p < 0.01; *** = p < 0.001; **** = p < 0.0001. Results are representative of two or more experiments.

Our prior studies demonstrated that CD103^+^ CD8 T cell formation in Panc02-SIY tumors is prevented by CD40L blockade at the time of tumor implantation. Since CD40-CD40L interactions play a critical role in the licensing of dendritic cells by CD4 T cells^1–3^, we designed experiments to determine whether the initial CD8 T cell interaction that results in CD103^+^ CD8 T cell formation is dependent on CD4 help. We implanted PK5L1940 tumors into untreated immunocompetent C57BL/6 mice, or into mice that were given a single dose of anti-CD4 depleting antibody 1 day prior to implantation (Fig. 4ai–ii). Successful depletion of CD4 T cells was confirmed by flow cytometry 7 days following tumor implantation (Supplementary Fig. S4a). As a comparison, we also included mice given anti-CD40L at the time of tumor injection to block CD103^+^ CD8 T cell formation in the tumor^11^. Implantation of PK5L1940 pancreatic adenocarcinoma tumors results in a detectable peripheral blood CD8 T cell response to SIY (Supplementary Fig. S4a), and while anti-CD40L treated mice had a significant decrease in the number of tumor antigen-specific T cells in the peripheral circulation, CD4 depletion did not impact the CD8 T cell response (Supplementary Fig. S4a-b). To evaluate whether CD4 depletion impacted tumor-specific CD103^+^ CD8 T cell formation, we examined T cells in the tumor environment. Consistent with our prior observations^11^, anti-CD40L treatment at tumor implantation did not impact the number of SIY-specific T cells, but significantly decreased the proportion of antigen-specific T cells that express CD103 (Supplementary Fig. S4c). CD4 depletion at tumor implantation did not affect the proportion of antigen-specific T cells in the tumor, or the proportion that express CD103 (Supplementary Fig. S4c). To confirm the effect of CD4 depletion on CD103^+^ CD8 T cell formation across a range of GEMM-derived tumor models, we tested the effect of CD4 depletion in a total of four different PK5L cell lines derived from Pdx-Cre^+/−^ Kras^(G12D)+/−^ Trp53^(R172H)+/−^ Luciferase-SIY^+^ mice. In each of these tumor models, CD4 depletion at tumor implantation did not affect CD103^+^ CD8 T cell formation (Fig. 4bi–iv). These data indicate that CD40L blockade at tumor implantation limits an initial CD8 T cell response required to form CD103^+^ CD8 T cells in the tumor. By contrast CD4 depletion does not impact the initial tumor antigen-specific CD8 T cell response in the peripheral blood or the ability to form CD103^+^ CD8 T cell populations in the tumor. One caveat is that CD4 depletion also removes T regulatory cells that may suppress T cell responses. To evaluate whether Treg depletion impacts CD103^+^ CD8 T cell formation, we treated mice with anti-CD25 1 day prior to cancer cell implantation. Treg depletion in established tumors can improve the function of CD8 T cell infiltrates, impact tumor growth, and response to subsequent therapies^36,37^. However, Treg expansion occurs progressively as tumors grow^37^ and the expansion of these cells has been shown to occur after the initial CD8 T cell expansion following tumor implantation^38^. We found that anti-CD25 treatment prior to implantation did not impact the initial peripheral blood SIY-specific T cell response to tumor implantation (Supplementary Fig. S5), and while it decreased the proportion of T regulatory cells in the tumor, it did not impact the accumulation of SIY-specific T cells in the tumor or the proportion of these cells that express CD103 (Supplementary Fig. S5). Together, these data suggest that tumor CD103^+^ CD8 T cell formation is CD40L-dependent but CD4 independent.

### Role of CD4 T cells and CD40L interactions in functional circulating tumor-specific CD8 T memory formation following tumor implantation

**Figure 5:**
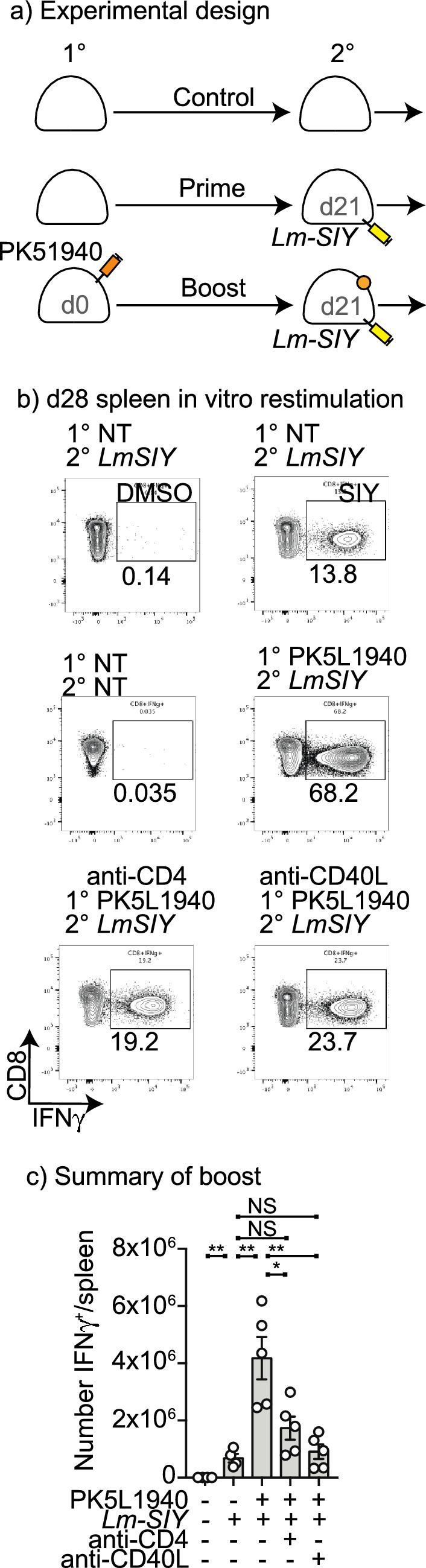
Decreased tumor-specific memory following anti-CD40L or anti-CD4 treatment at tumor implantation. (**a**) PK5L1940 pancreatic adenocarcinoma cells were injected sc. into C57BL/6 mice or C57BL/6 mice treated with anti-CD40L on d0, 1, and 2, or C57BL/6 mice treated with anti-CD4 on d-1. Control mice received no treatment. (**b**) Mice were treated with Lm-SIY on d21 and analyzed for SIY-specific T cells in the spleen on d28 by intracellular cytokine staining for IFNg following SIY peptide stimulation. (**c**) Quantitative analysis of SIY-specific cells in the spleen at d28.

Although primary responses to infectious agents can be unchanged in the absence of CD4 T cells, secondary responses are often decreased because of altered formation of CD8 T cell memory^39,40^. To assess this, we used a heterologous boost via IV administration of *Listeria monocytogenes* expressing the tumor-associated model antigen SIY (*Lm-SIY*). Due to the progressive growth of the primary tumor, the standard timescale of priming and boosting had to be compressed. As before, mice were treated with anti-CD40L or anti-CD4 at tumor implantation (Supplementary Fig. S6a), which represented the priming event, resulting in appropriate decreases in peripheral blood CD4 T cells with anti-CD4 treatment (Supplementary Fig. S6bi), no impact on CD8 T cell numbers (Supplementary Fig. S6bii), and decreased numbers of tumor-specific T cells with anti-CD40L treatment (Supplementary Fig. S6biii). These mice were then challenged with *Lm-SIY* 21 days following tumor implantation (Fig. 5a). In naïve mice that did not receive implantation of the tumor, *Lm-SIY* generated a strong primary response resulting in approximately 14% of CD8 T cells in the spleen making SIY-specific responses 7 days following injection (Fig. 5b). In mice previously implanted with SIY-expressing tumors the mice exhibited a boost response to *Lm-SIY* with up to 70% of all CD8 T cells being SIY-specific in the spleen. By contrast, mice treated with anti-CD4 or anti-CD40L at tumor implantation generated a significantly smaller secondary response (Fig. 5b-c). These data suggest that while CD4 depletion at tumor implantation does not impact CD103^+^ CD8 T cell formation, it does limit a secondary boost response at a distant site. Therefore, consistent with data in infectious disease models, CD4 T cells play a critical role in licensing CD8 T cells to differentiate into circulating memory populations. Therefore, there is a disconnect between the conditions needed to form Trm versus Tcm. Importantly, anti-CD40L treatment at tumor implantation limits formation of both Trm and circulating CD8 T cell memory populations.

### Role of CD40 and CD40L on CD8 T cells in Trm formation following tumor implantation

**Figure 6:**
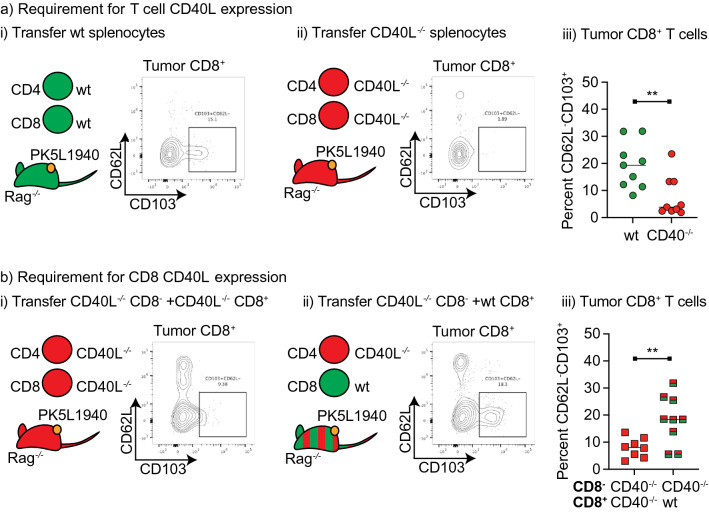
CD8 T cell can provide CD40L for Trm formation. (**a**) Rag^−/−^ mice were adoptively transferred with splenocytes from wt or CD40L^−/−^ mice, then following reconstitution, implanted sc. with PK51940 tumors. d14 tumors were harvested and analyzed for tumor infiltrating CD8 T cell phenotypes. Flow plots show representative CD103 expression in tumor CD8 T cells from mice reconstituted with (i) wt or (ii) CD40L^−/−^ splenocytes. (iii) Summary of CD103 expression in tumor T cells. (**b**) Rag^−/−^ mice were reconstituted with CD8-depleted CD40L^−/−^ splenocytes, along with purified CD40L^−/−^ CD8^+^ splenocytes, or purified wt CD8^+^ splenocytes. Following reconstitution, mice were implanted sc. with PK51940 tumors. d14 tumors were harvested and analyzed for tumor infiltrating CD8 T cell phenotypes. Flow plots show representative CD103 expression in tumor CD8 T cells from mice reconstituted with (i) CD40L^−/−^ CD8 T cells, or (ii) wt CD8 T cells. (iii) Summary of CD103 expression in tumor T cells. . Key: ** = p < 0.01. Results are the combination of two experiments.

The classic three cell model of DC licensing by CD4 T cells via CD40-CD40L interactions to permit full CD8 T cell activation requires CD40L expression by CD4 T cells^1–3^. Since in our models CD4 T cells are dispensable but CD40L is essential, it suggests the three-cell model may not be functioning. CD8 T cells themselves express CD40L on activation and have the capacity to ligate CD40 in the absence of CD4 help^41,42^. To establish whether CD40L on CD8 T cells can be sufficient to form CD103^+^ CD8 T cells, we first established a model for reconstitution of Rag^−/−^ mice with splenocytes from wild-type or CD40L knockout mice. T cells were allowed to reconstitute over 4 weeks, then mice were implanted with tumors (Fig. 6a). Analysis of tumor infiltrating cells suggested that mice lacking CD40L in their T cells were significantly impaired in their ability to form CD103^+^ cells in the tumor (p < 0.01) (Fig. 6ai–iii). To determine whether CD8 T cells could provide such signals independent of CD4 T cells, we reconstituted Rag^−/−^ mice with CD8-depleted splenocytes from CD40L knockout mice, as well as purified naïve CD8 T cells from either CD40L knockout or wild-type mice (Fig. 6b). In these reconstituted mice only the CD8 T cells have the potential to provide CD40L. The mice were implanted with tumors, and tumor infiltrating cells were analyzed as previously. Where only CD8 T cells have the capacity to express CD40L, this results in a significant restoration of CD103^+^ populations in the tumor (p < 0.01) (Fig. 6b). These data indicate that CD8 T cells are a sufficient source of CD40L to allow the formation of CD103^+^ CD8 T cells in tumors.

### Effect of CD40L blockade on systemic tumor immunity

**Figure 7:**
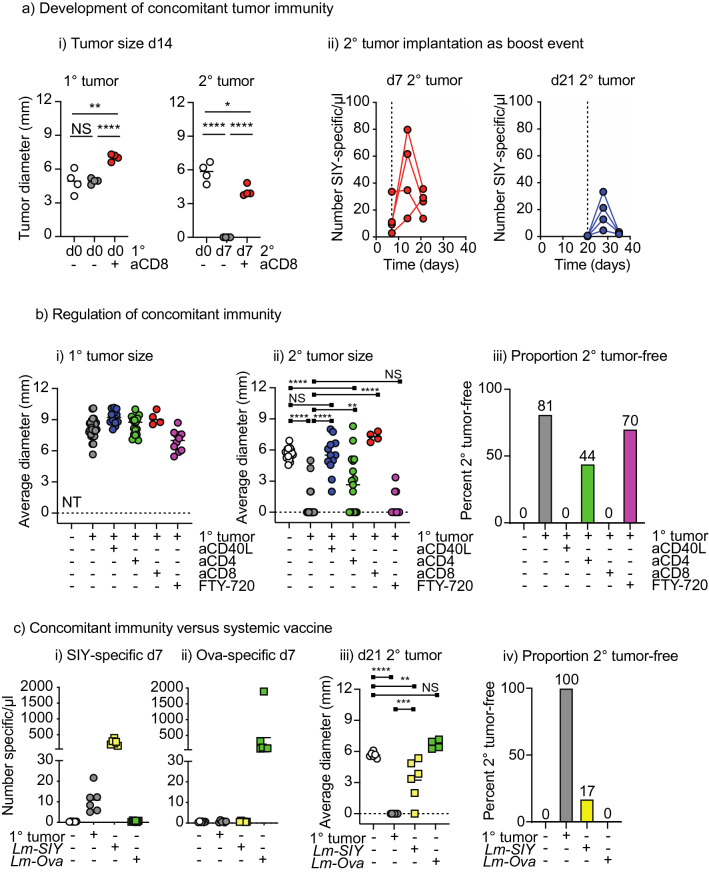
Concomitant tumor immunity is dependent on CD40L. (**a**)(i) Mice were implanted with a primary sc. PK5L1940 tumor on d0 and a secondary sc. tumor on the opposite flank at d0 or d7 (with and without CD8 depletion). Graphs show the size of the primary (1°) and secondary tumor (2°) at d14. (ii) Mice bearing a primary sc. PK5L1940 tumor were injected with a secondary tumor sc. on d7 or d21. The number of SIY-specific CD8 T cells were determined 0, 7 and 14d following secondary tumor implantation. (**b**) Mice were implanted with a primary sc. PK5L1940 tumor or no treatment at d0, and a secondary sc. tumor on d7. Groups of mice also received anti-CD40L on d0, 1, and 2, anti-CD4 on d-1, or anti-CD8 on d6. Additional groups received FTY-720 IP on days 6–11. Graphs show the size of the (i) primary (1°) and (ii) secondary tumor (2°) on d21. (iii) Proportion of mice secondary tumor free on d21. (**c**) Mice were implanted with a primary sc. PK5L1940 tumor or no treatment at d0 or were vaccinated with Lm-SIY or Lm-Ova IV on d0. Mice received a secondary sc. tumor on d7. Peripheral blood was analyzed for (i) SIY-specific T cells or (ii) ova-specific T cells on d7. (iii) Size of secondary tumors on d21. (iv) proportion of mice tumor-free on d21. Key: NS = not significant; * = p < 0.05; ** = p < 0.01; *** = p < 0.001; **** = p < 0.0001. Results are the combination of 4–5 experiments.

To understand the functional impact of circulating versus resident populations generated following tumor implantation on systemic responses, we established models of concomitant tumor immunity. Mice were implanted with primary PK5L1940 tumors on d0, and secondary PK5L1940 tumors on the opposite flank on the same day (d0), or with delayed implantation of the secondary tumor (d7). Simultaneously applied tumors grew equivalently, but a delay in tumor administration resulted in concomitant tumor immunity (Fig. 7ai). As would be expected, this concomitant tumor immunity is lost with CD8 depletion, though the second tumor is smaller than the primary tumor due to the shorter period of growth (Fig. 7ai). Secondary tumor implantation acted as a tumor-specific vaccine boost event, with the injection of cancer cells on d7 resulting in an expansion of circulating SIY-specific T cells (Fig. 7aii), and this could still be observed if the secondary tumor was implanted on d21, when circulating SIY-specific T cells have fallen below the limit of detection in the blood (Fig. 7aii). To determine whether this concomitant tumor immunity was affected by Tcm or CD103^+^ CD8 T cell formation, we repeated the experiment with either CD4 depletion at challenge to limit Tcm formation, or CD40L blockade to limit Tcm and CD103^+^ CD8 T cells. In the control group, primary tumor implantation limited secondary tumor growth compared to tumor implantation in naïve mice (p < 0.0001, Fig. 7bi–ii). CD40L blockade at primary tumor implantation prevented concomitant tumor immunity (Fig. 7bi–ii), with tumor growth no different from naïve mice (Fig. 7bi–ii). Concomitant immunity was only partially impacted by CD4 depletion (Fig. 7b), with significantly larger tumors than mice with primary tumors and no CD4 depletion (p < 0.0001), but significantly smaller tumors than naïve mice (p < 0.01) and approximately half of the mice rejected the second tumor (Fig. 7biii). As before, CD8 T cells were essential for concomitant immunity, but interestingly concomitant immunity was not impacted by FTY720 treatment at the time of second tumor implantation (Fig. 7bi–iii), suggesting that recirculation is not necessary for distant tumor rejection after tumor-specific T cells are formed following primary tumor injection.

Given the limited need for CD4 T cells to generate concomitant immunity, we compared the ability of a potent vaccine versus tumor implantation to protect against distant tumor challenge. Mice were either given a tumor on one flank or IV *Listeria monocytogenes* expressing the model tumor antigen SIY or the irrelevant model antigen Ova, then challenged with a tumor on the opposite flank. *Listeria* vaccination resulted in a dramatically higher circulating T cell response than tumor challenge (Fig. 7ci–ii). However, while *Listeria* vaccination with tumor-associated antigens was able to slow the growth of subsequently implanted tumors, compared to concomitant immunity generated by primary tumor implantation *Listeria* vaccination was significantly less able to prevent tumor growth (p < 0.01) (Fig. 7c). Together, these data indicate that despite being a much more potent vaccine for T cell expansion, single-antigen vaccine-mediated systemic expansion of T cells is less effective in protecting against tumor challenge than concomitant immunity. These data suggest that tumor implantation generates tumor-specific T cells that while less frequent in the circulation, are more effective in protecting against tumor challenge.

## Discussion

We generated a novel panel of pancreatic cancer cell lines with stable expression of a model antigen and observe that CD103^+^ CD8 T cell formation is dependent on antigen-specific interactions in the lymph node but occurs independently of CD4 T cell help. In addition, we observe that cells with a Trm phenotype are actively engaging with antigen in the tumor environment and depend on direct interaction to sustain the tumor T cell niche. We observe that the same CD40L-dependent signals are necessary to mediate concomitant tumor immunity, and that CD4 T cells are not essential for concomitant tumor immunity. These data suggest that CD103^+^ CD8 T cell formation and tumor-specific concomitant immunity is a CD4 T cell independent event and does not require the two-factor authentication critical to generate classical circulating T cell memory. An important caveat in this work is that the tissue residency of the CD103^+^CD39^+^ CD8 T cells has not been demonstrated. Ongoing work aims to define the relative movement of these cell populations as compared to other T cell populations that infiltrate the tumor, given that most non-resident T cells dynamically move through tissues and back to the peripheral blood via recirculation^43^. Additionally, transcriptional profiling may be necessary to define whether the CD103^+^CD39^+^ CD8 T cells that infiltrate tumors in the presence or absence of CD4 T cells are transcriptionally aligned with Trm or with T cells of other differentiation pathways.

While CD103^+^ CD8 T cells are found in the tumor, our data makes it clear that these cells originate following conventional interactions with antigen in the lymph nodes. In LCMV infection models, CCR7 expression by T cells and DC is important, but not essential^44^, and central memory formation in response to LCMV infection can be improved where mice have CCR7^−/−^ T cells^45^. However, in tumor models, MHC mismatched (allogenic) tumors that are ordinarily rejected in wild type mice are able to grow in CCR7^−/−^ mice^46^ and CCR7 expression by T cells was essential for tumor control^46^. These data are consistent with those of Mani et al., who demonstrate a role for DC preconditioning of CD8 T cells in secondary lymphoid organs to form Trm populations^47^. Together, these data demonstrate a strong dependence on lymph node-DC interactions for tumor Trm formation and functional tumor rejection.

One explanation for CD4 independence is that CD103^+^ CD8 T cells form in response to higher affinity antigens that can be CD4 independent. In tumors, Trm enrich for tumor specificity^7,8^ and can also enrich for exhaustion markers that are associated with higher affinity T cells^8^. However, there is mixed evidence linking Trm to higher affinity antigens in infectious disease models^30,48,49^. Following influenza infection of the lung CD4 T cells have been shown to be essential for Trm formation^50^, suggesting that the role of CD4 T cells and the affinity of Trm may be model-dependent. Interestingly in this latter model, regulating T-bet in the unhelped CD8 T cells restored Trm differentiation in the absence of CD4 T cells, suggesting that early differentiation features of CD8 T cells, impacted by their inflammatory environment^51^, may dictate CD4 dependence and Trm differentiation.

Using altered peptide ligands to stimulate T cells, Stark et al*.* demonstrated that CD40L expression on CD8 T cells is best induced with higher affinity ligands^52^, consistent with data suggesting that this CD4 independent effect may only occur to higher affinity antigens, resulting in Trm being enriched for high affinity antigens in the weakly inflammatory setting of tumor implantation. We observe that CD8 T cells may be a sufficient source of CD40L to activate CD40-expressing cells and in turn support CD8 differentiation into CD103^+^ cells in the tumor environment. Based on the literature, we assume that the CD8 T cells are licensing DC for DC activation, in place of CD4 T cells^1–3^; however we have not demonstrated a direct activation of dendritic cells or a role for dendritic cells in this study. However, these results are consistent with prior work in naïve and memory CD8 T cell populations showing direct activation of dendritic cells via CD8-expressed CD40L^41,42^. Pancreatic tumors engineered to overexpress an immunogenic antigen were shown to be rejected when implanted subcutaneously, but to mostly grow when implanted orthotopically into the pancreas^53^. Rejection of subcutaneous tumors and control of a subset of orthotopic pancreatic tumors was correlated to the degree of tumor-specific T cell expansion following implantation and providing DC maturation signals via CD40 at the time of tumor implantation prevented orthotopic pancreatic tumor growth^53^. Importantly, in these models rejection of subcutaneous tumors was independent of CD4 T cells, but by titrating the orthotopic tumor dose down so that the tumor is rejected in wild type mice, this rejection is both CD8 and CD4 dependent^53^. These data suggest that dendritic cell maturation is a critical factor determining the protective immune response that is established following tumor implantation, and that CD4 T cell help becomes a critical factor only where DC maturation signals are absent, or at very low antigen doses.

In a range of vaccination models, Trm have been shown to share clonal origins with Tcm^54^. While Trm generate their characteristic phenotype on tissue entry, and Trm can form from Tcm^55^, they may be pre-programmed towards Trm differentiation much earlier in development^56^. Interestingly, data suggests that the earliest CD8 T cells at an infection site have many of the features of Trm^57^. Similarly, T cells lacking the transcriptional regulator Hobit are unable to form Trm but form Tcm in normal proportions^58^, indicating that transcriptional differences direct Trm along their path and away from Tcm differentiation. Early exposure of CD8 T cells to antigen has been shown to be critical to Trm generation, while Tcm formation was not impaired by delaying exposure to antigen^59^. These data are consistent with our observations that CD40L blockade only at tumor implantation is sufficient to block CD103^+^ CD8 T cell development.

Peripheral vaccination has been shown by others to generate Trm that can protect against tumor implantation^60,61^ and Trm formed by tumor implantation are protective against further tumor challenge^62^. In our models, tumor growth was poorly affected by prior vaccination with a strong systemic vaccine. This is consistent with our previous experience where established pancreatic tumors are poorly impacted by vaccination alone^14,63^, in contrast to more immunogenic tumor models that are protected^64^. In each of these systems the vaccine was designed around a strong immunodominant target antigen. While tumor implantation is still subject to immunodominance it is possible that a range of subdominant antigens contribute to anti-tumor immune responses but are absent from single target vaccines. A wide range of tumor antigen vaccine platforms have been developed and translated to patients, yet the clinical response has been limited^65^. Together our data are consistent with Trm formation in tumors occurring because of exposure to higher affinity antigens that permit CD4 independent activation of CD8 T cells via their own CD40L. We propose that where strong adjuvant signals are present, Tcm formation may dominate, and these are less able to protect against subsequent tumor challenge despite their increased number. We conclude that approaches aiming to achieve systemic tumor surveillance following vaccination should be focused on scenarios that promote Trm formation, even if this is at the expense of high-level T cell expansion.

## Methods

### Animal studies

All animal experiments were performed in compliance with the National Institutes of Health Guidelines and were approved by the Earle A. Chiles Research Institute Institutional Animal Care and Use Committee. Reporting methods follow ARRIVE guidelines^66^. C57BL/6 (Stock No: 000664), R26^LSL-LSIY^ (Stock No: 009044), *Kras*^*LSL-G12D/*+^ (Stock No: 008179), *Pdx1-Cre* (Stock No: 014647), CCR7^−/−^ (Stock No: 006621) Rag1^−/−^ (Stock No: 002216), and CD40L^−/−^ mice (Stock No: 002770) were purchased from The Jackson Laboratory. *Tp53*^*LSL-R172H/*+^ (Stock No: 01XM2) were obtained from the Frederick National Laboratory for Cancer Research, and backcrossed n > 6 onto the C57BL/6 background before generating KPC or KPC-LSIY mice. Nur77^GFP^ reporter mice^29^ were kindly provided by Dr. Weinberg (Earle A. Chiles Research Institute, Portland, OR). 2C transgenic mice that express a T cell receptor specific for the SIYRYYGL (SIY) peptide presented on H2K_b_ were kindly provided by Dr. Gajewski (University of Chicago, Chicago, IL). OT-I transgenic mice that express a T cell receptor specific for the SIINFEKL peptide presented on H2K_b_, and POET mice that express ovalbumin in the prostate^24^ were kindly provided by Dr. Redmond (Earle A. Chiles Research Institute). Kaede transgenic mice were kindly provided by Dr. Lund at Oregon Health & Science University^34^. Kaede transgenic mice were crossed with CCR7^−/−^ mice in house to generate Kaede CCR7^−/−^ animals as previously described^67^.

To monitor tumor-related outcome in *Pdx1-Cre Kras*^*LSL-G12D*^* Tp53*^*LSL-R172H*^ and *Pdx1-Cre Kras*^*LSL-G12D/*+^ *Tp53*^*LSL-R172H/*+^ R26^LSL-LSIY^ mice, animals were monitored for body condition, and animals were euthanized when an animal lost more than one point of body condition, more than 15% of body weight, or had other morbidities that indicate pain or distress such as hunched posture. We also regularly palpated for tumors and monitored for ascites, which was typically accompanied by hunched posture. Palpation informs as to the presence of a tumor but is not an endpoint of itself. In prior studies using *Pdx1-Cre Kras*^*LSL-G12D*^* Tp53*^*LSL-R172H*^ mice the emergence of ascites closely predicted mortality^19^, so to avoid death as an endpoint, mice were euthanized by gradual CO_2_ asphyxiation and cervical dislocation according to AVMA guidelines when ascites developed. The presence of tumors in the pancreas and/or elsewhere was confirmed by necropsy. Tumors and tissues with visual evidence of macrometastases were collected for histologic confirmation. Briefly, formalin-fixed tumors and tissues were paraffin embedded and 5 µM sections were H&E stained and digitally scanned using a Leica SCN400 whole slide scanner.

### Cell line isolation and characterization

To generate cell lines primary tumors were surgically isolated from GEMM mice with established pancreatic masses. Tumors were surgically dissected into 2 mm fragments and seeded to collagen-coated 24 well plates for 30 min in minimal media. Once fragments were attached to plastic, the wells were gently filled with media (RPMI 1640 media supplemented with 10% FBS, 100 U/mL/100 µg/mL Penicillin/streptomycin, 10 mM HEPES, 1 × MEM non-essential amino acids, 1 mM sodium pyruvate, 55 µM µ-mercaptoethanol), and cultured for 5–7 days with regular media changes until cancer cell expansion from the base of the tumor fragments was detectable in some wells. Cancer cells were harvested by trypsinization and expanded through repeated passage into homogeneity. Cell lines were denoted by their genetic features as PK5 (PDX-Cre, Kras, p53) or PK5L (PDX-Cre, Kras, p53, Luciferase-SIY) plus a numerical identifier, such as PK5L1940. PK51975B-Ova cells were generated by stable transfection of a plasmid encoding a GFP-SIINFEKL fusion protein as previously described^11^. The Panc02-SIY pancreatic adenocarcinoma line expressing the model antigen SIY was kindly provided by Dr. Weichselbaum at the University of Chicago. For bioluminescent imaging, mice received an i.p. injection of 150 mg/kg D-Luciferin (Gold Biotechnology) 10 min prior to imaging. Mice were anesthetized and placed in the MuriGlo chamber (XStrahl, Atlanta, GA) and 3D images were acquired using Muriplan (XStrahl).

### CRISPR knockout of direct antigen presentation

Beta 2 microglobulin (B2M) knockout of PK5L1940 cell lines were generated as previously described^14^. Briefly, cells were transfected using Alt-R S.p. Cas9 Nuclease 3NLS (Integrated DNA Technologies, Coralville, IA), Alt-R CRISPR-Cas9 tracrRNA ATTO 550 (Integrated DNA Technologies) vendor-designed Alt-R CRISPR-Cas9 crRNA guide RNAs, with Lipofectamine CRISPRMAX Cas9 Transfection Reagent (Thermo Fisher Scientific, Waltham, MA) in Opti-MEM I Reduced Serum Medium (Thermo Fisher). Three gRNAs were tested for each target and pure populations were isolated from five cells based on ability to upregulate MHC-I in response to IFNg stimulation isolated using a BD FACSAria II cell sorter. Gene knockout was confirmed by qPCR for B2M. To evaluate the ability of these cells to directly prime T cells, cells were left untreated or pre-treated with IFNg for 24 h to upregulate MHCI, then cocultured with CD8 T cells purified from OT1 (SIINFEKL-specific TCR transgenic) mice, or 2C (SIY-specific TCR transgenic mice) using an EasySep™ Mouse CD8 + T Cell Isolation Kit (Stemcell, Vancouver, Canada). T cells were harvested after 48 h and analyzed for expression of CD69 by flow cytometry.

### Tumor formation and concomitant tumor immunity models

To evaluate tumor formation, 0.2 × 10^6^ cells from PK5 and PK5L cell lines were resuspended in 50 mL of PBS and subcutaneously implanted into the flanks of 6–12-week-old C57BL/6 mice, or orthotopically following surgical exposure of the pancreas. For concomitant tumor immunity models, mice were implanted with tumors simultaneously on both flanks, or the tumor was implanted on the second flank following a 7-day delay. To deplete cell populations in vivo, mice were injected with 200 µg of anti-CD8b antibodies (BioXCell, Branford, CT clone 53–5.8), anti-CD4 (BioXCell clone GK1.5), or anti-CD25 (BioXCell clone PC-61.5.3) intraperitoneally one day prior to tumor implantation. Anti-CD40L (BioXCell clone MR1) antibodies were delivered i.p. as 3 daily 250 µg doses starting at the day of tumor implantation to establish tumor implant tolerance. To block recirculation, FTY720 was obtained from Cayman Chemical Company (Ann Arbor, MI) and was administered at 1 mg/kg/day intraperitoneally, starting 1 day prior to tumor implantation for a total of 5 consecutive days.

### L. monocytogenes vaccinations

*L. monocytogenes* strains used for these studies, *ΔactA* ActA-OVA (*Lm*-Ova, expressing the class I-K^b^ restricted OVA_257–264_ epitope)^68^ and *ΔactA* ActA-SIY (*Lm*-SIY expressing the class I-K^b^ restricted SIYRYYGL epitope)^68^, were grown to stationary phase in brain–heart infusion broth, washed in PBS, and 1 × 10^7^ CFU injected intravenously (retro-orbital route) in 200μL total volume. When priming responses were analyzed, spleens from infected mice were harvested after 7 days and processed for flow cytometry as described below. When prime/boost responses were analyzed, mice were boosted 21 days after the priming and spleens harvested and processed 7 days later.

### Tumor processing

Tumor processing was performed as recently described^67^. Briefly, following dissection into small fragments, tumors were transferred into C tubes from Miltenyi Biotec containing enzyme digest mix with 250U/mL collagenase IV (Worthington Biochemical, #LS004188), 30U/mL DNase I (Millipore-Sigma, #4536282001), 5 mM CaCl_2_, 5% heat inactivated FBS and HBSS. Tissue was dissociated using a GentleMACS tissue dissociator from Miltenyi Biotech. This was followed by incubation at 37 °C for 30 min with agitation and enzymatic reactions were quenched using ice cold RPMI containing 10% FBS and 2 mM EDTA. Single cell suspensions were filtered through 100 µm strainers to remove macroscopic debris. Cells were washed and counted for flow cytometry.

### Flow cytometry

For staining, 2 × 10^6^ cells were stained with Zombie Aqua Viability Dye from BioLegend (#423,102) in PBS for 10 min on ice, then Fc receptors were blocked with α-CD16/CD32 antibodies from BD Biosciences (2.4G2) for an additional 10 min. After centrifugation, the supernatant was removed and cell were stained with a surface antibody cocktail containing in FACS buffer (PBS, 2 mM EDTA, 2% FBS) and Brilliant Stain Buffer Plus from BD Biosciences (#566,385) for 20 min on ice. Antibodies were purchased from the following vendors: BioLegend; CD8α-BV650 (53–6.7), CD103 BV711 (2E7) CD39 PE-Cy7 (Duha59), CD44 APC-Cy7 (IM7), CD62L BV605 (Mel14), CD4 Alexa488 (RM4-5), CD4 APC-Cy7, CD25 BV605 (PC61), CD90.2 Alexa 700 (30-H12), Ly6C PerCP-Cy5.5 (HK1.4), Ly6C BV711, CD11b PE-dazzle (M1/70), Thermo Fisher Scientific; CD103 APC/Alexa 647 (2E7), and CD69-PE-Cy7 BD Biosciences; CD3 Alexa 700 (17A2), CD45-BV786 (30-F11), MHCI PE (AF6-88.5.5.3).

To quantify antigen-specific CD8 T cells in the tumor and peripheral blood, PE-conjugated Kb- SIYRYYGL pentamers were purchased from Proimmune (Sarasota, FL). SIINFEKL-Kb tetramers were obtained from the NIH Tetramer Core Facility at Emory University (Atlanta, GA). After surface staining, cells were washed in FACS buffer and fixed for 20 min on ice with Fixation/Permeabilization Buffer from BD Biosciences (#554722). All samples were resuspended in FACS buffer and acquired on a BD Fortessa flow cytometer. Data were analyzed using FlowJo software from Tree Star, v10.7.

Quantitative assay of cell depletion or antigen-specific cell numbers in the peripheral blood were measured using a whole blood bead assay as previously described^69^. Briefly, whole blood was harvested into heparin tubes, and stained directly with fluorescent antibody cocktails along with MHC-antigen multimers where appropriate. AccuCheck fluorescent beads (Invitrogen) were added to each sample, then red blood cells were lysed with BD FACS lysing solution (BD Biosciences), and samples analyzed on a BD LSRII flow cytometer. We determined the absolute number of cells in the sample based on comparing cellular events to bead events (cells/µL).

To measure splenocyte T cell responses by intracellular cytokine stimulation (ICS), spleens were harvested 7 days following treatment and cell suspensions were stimulated with 2 µM of SIY peptide (SIYRYYGL), Ova peptide, (SIINFEKL), or DMSO vehicle in the presence of brefeldin A for 4 h at 37 °C as previously described^68^. Stimulated cells were washed and stained with CD4-FITC and CD8-PerCP Cy5.5, then fixed and permeabilized using a BD Cytofix/Cytoperm plus kit (BD Biosciences) and frozen at − 80 °C. For analysis cells were thawed and intracellularly stained with IFNγ-APC. Cells were washed and analyzed on a BD LSRII Flow Cytometer and the data was interrogated using BD FACSDiva (BD Biosciences) and FloJo (Tree Star, Ashland, OR).

### Mixed reconstitution chimeras

Rag1^−/−^ recipient mice were adoptively transferred with 5 × 10^5^ wt or knockout splenocytes and allowed to reconstitute for 4 weeks prior to further study. For mixed reconstitution, mice were reconstituted with a 1:1 mix of each population, with a total transferred cell population of 5 × 10^5^ splenocytes. For adoptive transfer where the CD40L genotype of the CD8 T cells was different from the other splenocytes, CD40L^−/−^ donor splenocytes were sorted using magnetic beads to deplete CD8 T cells using an easySep™ Mouse CD8a Positive Selection Kit II (Stemcell, Vancouver, Canada) and wt or CD40L^−/−^ donor splenocytes were enriched for CD8 T cells by negative selection from other populations using an EasySep™ Mouse CD8 + T Cell Isolation Kit (Stemcell). In this way both the CD8-depleted CD40L^−/−^ donor splenocytes and the wt or CD40L^−/−^ donor CD8^+^ splenocytes are negatively selected. 1 × 10^5^ sorted CD8^+^ T cells from wt or CD40L^−/−^ donor spleens were added to 5 × 10^5^ CD8-depleted CD40L^−/−^ donor splenocytes to reconstitute Rag1^−/−^ recipient mice where no T cells, or only CD8^+^ T cells had the potential to express CD40L.

### Statistics

Data were analyzed and graphed using Prism from GraphPad Software (v9.0). Individual data sets were compared using Student’s T-test and analysis across multiple groups was performed using one-way ANOVA with individual groups assessed using Tukey’s comparison. Kaplan Meier survival curves were compared using log-rank tests.

## Supplementary Information

Supplementary Information.

## Data Availability

All data is present in the manuscript and supplemental figures. Raw data files are available on request to Michael Gough (michael.gough@providence.org).
