# Supplementary material for: Tumor resident memory CD8 T cells and concomitant tumor immunity develop independently of CD4 help
Source: Sci Rep. 2023 Apr 18;13:6277. doi: 10.1038/s41598-023-33508-1 (PMC10113239; doi:10.1038/s41598-023-33508-1)
Supplement: Supplementary file 1 — Supplementary Information. [file 41598_2023_33508_MOESM1_ESM.docx]

## Supplemental figures

### Figure S1. Vaccination of wt, R26LSL-LSIY, KPC-LSIY, and POET mice.

Mice of the indicated genotypes were left untreated or vaccinated with Lm-SIY or Lm-OVA, with antigen specific responses to a) SIY, or b) SIINFEKL measured in the blood 7 days post-vaccination. a-b) Genotypes are marked in blue, and vaccines given are marked in red. b-c) Mice were treated with vaccines as in a-b), with a boost given 42 days later. 7 days post-boost, splenocytes were harvested and the percent of cells specific for c) SIY, or d) SIINFEKL was determined by IFNg^+^ intracellular cytokine staining. Data are represented as means +/- SEM, with statistical significance performed by student’s T test. Key: NS = not significant; * = p<0.05; ** = p<0.01; *** = p<0.001; **** = p<0.0001.

### Figure S2. Differentiation of T cells that are not SIY-specific in tumor and lymphoid organs

a) Analysis of SIY-specific CD8 T cells in the peripheral blood following tumor implantation. i) representative flow cytometry of CD3+ T cells showing SIY-specific CD8 T cell populations with no tumor implantation and with PK5L1940 tumor implantation. ii) quantitation of the number of SIY-specific CD8 T cells in the peripheral blood in mice left untreated as compared to mice implanted with PK5L1940 tumors over time following tumor implantation. b) PK5L1940 tumors were implanted into C57BL/6 mice and tumors, tumor-draining lymph node (TDLN), non-draining lymph node (NDLN), and spleen were analyzed for CD8 T cell phentotypes at d14. Figure 2 shows the result for SIY-specific T cells. These plots show matched T cells that are not SIY-specific, showing i) CD62L and Ly6C expression on CD8 T cell populations of unknown specificity in the lymphoid organs versus the tumor, ii) quantification of these populations across samples – each symbol represents 1 tumor. Key: *** = p<0.001; **** = p<0.0001.

### Figure S3. Gating strategy to identify SIY-specific and Trm populations

a) PK5L1940 tumors were implanted into Nur77-GFP mice and tumor infiltrating T cells were analyzed by flow cytometry. Representative flow plots identifying the SIY-specific T cell population of infiltrating CD8 T cells by SIY-pentamer staining, the CD103+ and CD103+CD39+ subpopulations, and for each their Nur77-GFP expression was determined. b) Graphs show a matched experiment with Panc02-SIY tumors in Nur77-GFP mice, graphs show i) Percent Nur77-GFP+ and ii) MFI of Nur77 in each group. . Key: NS = not significant; * = p<0.05; ** = p<0.01; *** = p<0.001; **** = p<0.0001.

### Figure S4. Effect of CD40L blockade or CD4 depletion on peripheral blood and tumor CD8 T cell responses to tumor implantation.

a) PK5L1940 pancreatic adenocarcinoma cells were injected into C57BL/6 mice, C57BL/6 mice treated with anti-CD40L on d0, 1, and 2, or C57BL/6 mice treated with anti-CD4 on d-1. Graphs show representative flow cytometry of peripheral blood T cells 7d following implantation. b) Summary of quantitative analysis of peripheral blood i) CD4; ii) CD8; and iii) SIY-pentamer+ CD8 T cells per µl blood. C) T cell infiltration of PK5L1940 tumors derived from Pdx-Cre+/- Kras(G12D)+/- Trp53(R172H)+/- Luciferase-SIY+ mice implanted into C57BL/6 mice or C57BL/6 mice treated with anti-CD4 on d-1. Graphs show tumor the proportion of CD90.2+ T cells that are i) CD4; ii) CD8; iii) the proportion of CD8 T cells that are SIY-pentamer+ T cells, and iv) the proportion of SIY-pentamer+ T cells that express CD103. Key: NS = not significant; * = p<0.05; ** = p<0.01; *** = p<0.001; **** = p<0.0001.

### Figure S5. Effect of CD25 depletion on Trm formation

PK5L1940 tumors were implanted into C57BL/6 mice left untreated or treated with anti-CD25 1 day prior to tumor implantation. a) Absolute numbers of CD8 T cells and SIY-specific T cells in the peripheral blood 7 days following tumor implantation. b) Tumor infiltrating T cells were analyzed by flow cytometry. Graphs show the proportion of CD4 T cells that are CD25+FoxP3+ T regulatory cells, the SIY-specific T cell population, and the CD103+ subpopulation of SIY-specific T cells.

### Figure S6. Initial T cell response to tumor implantation prior to boosting

a) PK5L1940 pancreatic adenocarcinoma cells were injected into C57BL/6 mice, C57BL/6 mice treated with anti-CD40L on d0, 1, and 2, or C57BL/6 mice treated with anti-CD4 on d-1. Control mice received no treatment. b) Summary of quantitative analysis of peripheral blood d7 following tumor implantation, showing the number of i) CD4; ii) CD8; and iii) SIY-pentamer+ CD8 T cells per µl blood. Key: NS = not significant; * = p<0.05; ** = p<0.01; *** = p<0.001; **** = p<0.0001.
